# Supplementary material for: Assessment of Biofilm Formation and Anti-Inflammatory Response of a Probiotic Blend in a Cultured Canine Cell Model
Source: Microorganisms. 2024 Nov 11;12(11):2284. doi: 10.3390/microorganisms12112284 (PMC11596120; doi:10.3390/microorganisms12112284)
Supplement: Supplementary file 1 [file microorganisms-12-02284-s001.zip › microorganisms-3227769-supplementary.pdf]

Supplementary figure

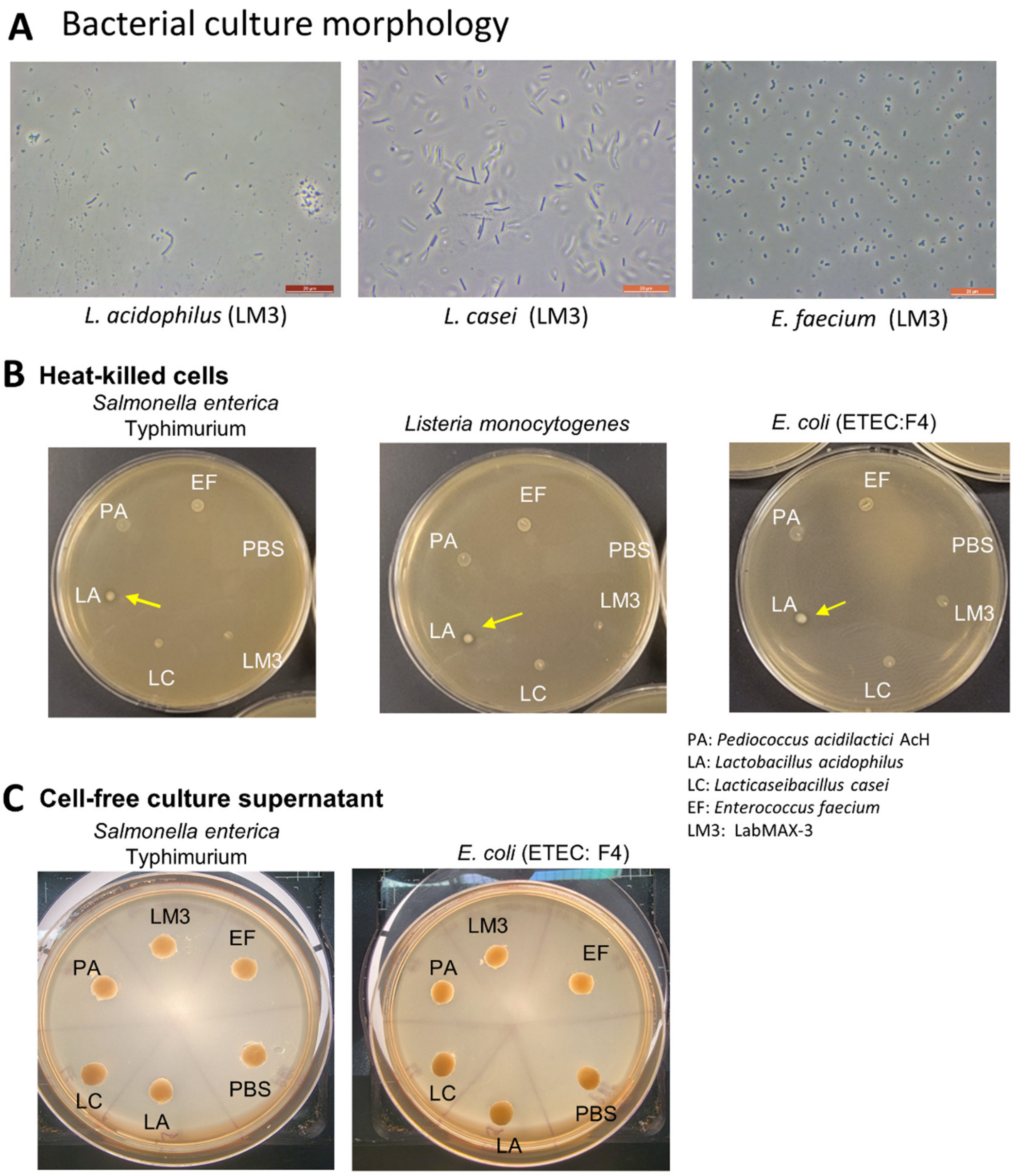

Figure S1. Probiotic bacterial morphology and antimicrobial activity. Probiotic culture isolates morphology (A) and antimicrobial activity of heat-killed (B), and cell-free culture supernatants (C) against pathogens. In panel A, the morphology of probiotic culture isolates was examined under a phase contrast microscope. In Panel B, heat-killed cells (10 µl) were deposited on an MRS agar plate, which was overlaid with test strains suspended in TSA soft agar (0.8%) preparation suspended in test pathogens. Zone of Inhibition was produced only by heat-killed LA cells. In Panel C, cell-free culture supernatants (40 µl) were deposited on blotting paper discs on a lawn of pathogens on the BHI agar surface.
